# Supplementary material for: Giant field enhancement in high-index dielectric subwavelength particles
Source: Sci Rep. 2017 Apr 7;7:731. doi: 10.1038/s41598-017-00724-5 (PMC5429612; doi:10.1038/s41598-017-00724-5)
Supplement: Supplementary file 1 — Supplementary info [file 41598_2017_724_MOESM1_ESM.pdf]

Giant field enhancement in high-index dielectric subwavelength particles

Supplementary Information

Polina Kapitanova[1,+], Vladimir Ternovski[2,+], Andrey Miroshnichenko[3,+,*],
Nikita Pavlov[1], Pavel Belov[1], Yuri Kivshar[1,3], Michael Tribelsky[2,4]

[1]*ITMO University, St. Petersburg 197101, Russia*

[2]*Lomonosov Moscow State University, Moscow 119991, Russia*

[3]*Nonlinear Physics Centre, Research School of Physics and Engineering, Australian National University, Canberra ACT 2601, Australia*

[4]*National Research Nuclear University MEPhI (Moscow Engineering Physics Institute), Moscow 115409, Russia*

[*]E-mail: andrey.miroshnichenko@anu.edu.au

[+]these authors contributed equally to this work

A. Experimental study of the distilled water permittivity

The experimental permittivity data for pure water at the standard atmospheric pressure are known for a wide range of frequencies and temperatures [1, 2]. In our study we used commercially available distilled water. The complex permittivity of water (especially its imaginary part) depends substantially on the composition and concentration of dissolved microimpurities. Since for the samples of water employed in our experiment these quantities were not known, we measured the complex permittivity of the samples in the domains of the water temperature and radiation frequencies used in out experiments and checked the obtained results against the data presented in Refs. [1, 2].

The photograph of the experimental setup is demonstrated in Suppl. Fig. 1 (a). The setup consists of a Vector Network Analizer (VNA), a Dielectric Assessment Kit (DAK) probe, DAK Software and a thermometer. The DAK offers high-precision dielectric parameter measurements (permittivity, permeability, conductivity, loss tangent) of liquids over the very broad frequency range from 10 MHz to 67 GHz for applications in the electronic, chemical, food, and medical industries [3]. The DAK probe was place in the cavity with the distilled water and connected to the VNA that measured the complex reflection coefficient (S_{11}) at the probe end. The measured reflection coefficient was then converted to the complex permittivity of the water under the test using DAK Software. The DAK software is based on a very precise and fast method, formulated and published by Ellison [1]. To control the water temperature during the measurements a thermometer was used.

The obtained real and imaginary parts of the distilled water permittivity measured in the frequency range of 0.5 - 4 GHz for the temperature range of 20°-90° C are presented in Suppl. Fig. 1 (b) and (c), respectively. The measured data agreed well with the target data taken from Kaatze [2].

B. Experimental study of the field distribution inside the cylinder

To measure the field distribution inside the cylinder the near-filed scanning technique is used. A schematic view of the experimental setup is shown in Suppl. Fig. 2. A rectangular horn antenna (TRIM 0.7518 GHz; DR) connected to the first (transmitting) port of a VNA Agilent E8362C is employed to approximate a plane wave excitation. The antenna is placed at the 2 m distance from the cylinder. A coaxial cable with an electric or magnetic probe attached to its end is connected to the second (receiving) port of the VNA and fixed at an arm

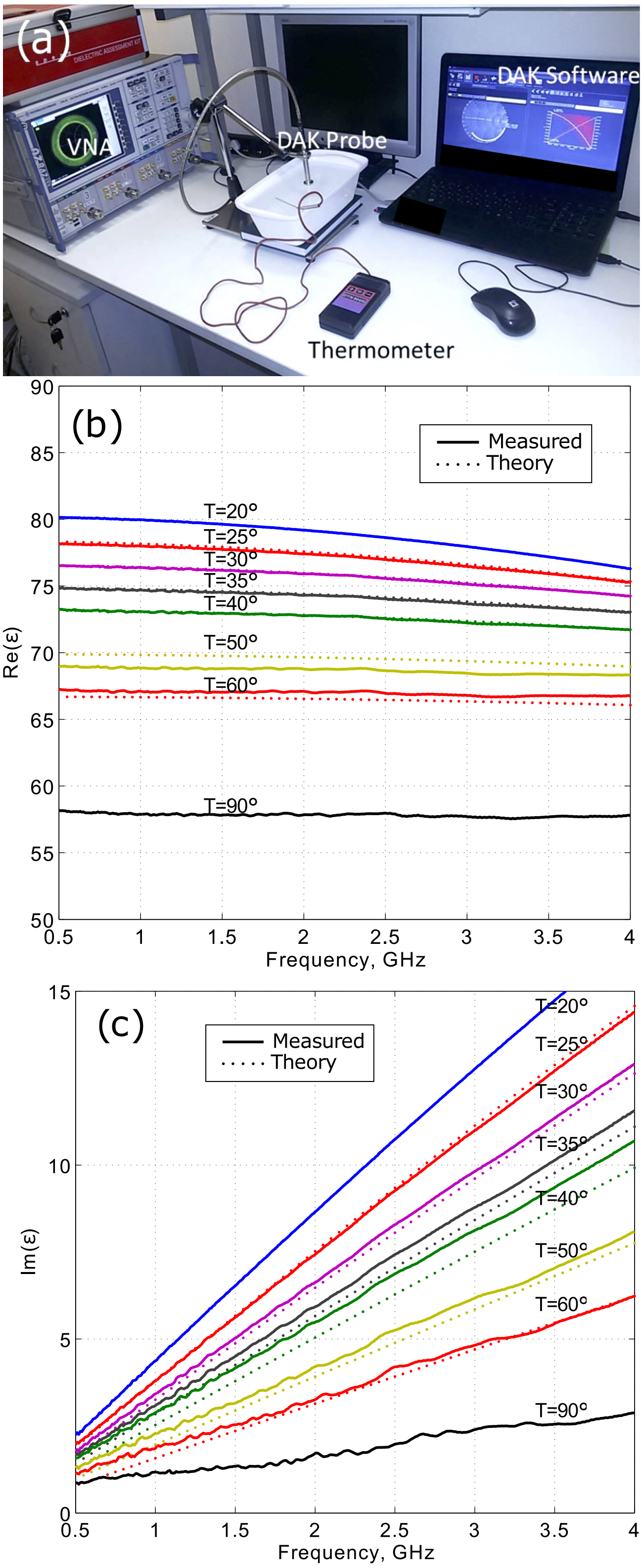

SUPPL. FIG. 1: (a) Experimental setup of water permittivity measurement; (b) the measured real and (c) imaginary parts of the permittivity. The solid lines represent the measured data, the dashed lines show the target data taken from Kaatze [2].

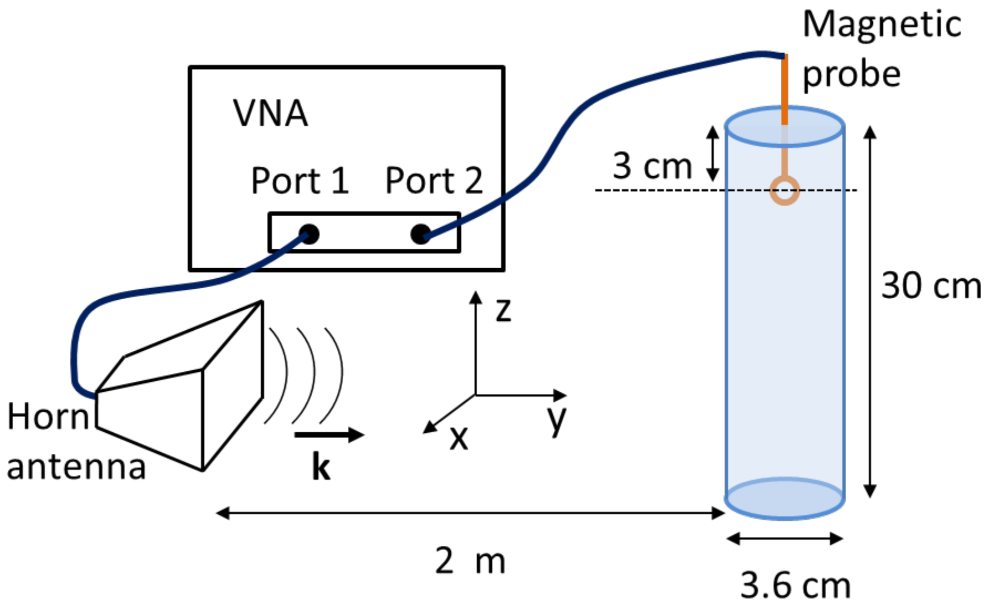

SUPPL. FIG. 2: Schematic view of near-field measurement setup.

of an automatic, mechanical scanner (not shown in Suppl. Fig. 2). During the measurements the scanner arm with the probe is automatically moved in the x-y plane at 3 cm under the surface of water with a predetermined step. The VNA measures the complex value of the transmission coefficient S_{21}, which corresponds to the electric or magnetic field intensity, depending on the type of the attached probe. In order to measure the electric filed distribution inside the cylinder the open end coaxial cable is used. Since the electric field enhancement occurs much smaller than that for the magnetic field, in what follows only the results for the magnetic field are discussed. To measure the magnetic field distribution inside the cylinder, we use a small loop antenna as a probe [4]. The measurements are performed for both of the two independent polarizations: TE and TM. To change the polarization the antenna orientation was changed.

For the TE polarization the magnetic field inside the cylinder has a single non-zero component: H_z. To measure this field the receiving loop antenna was set horizontally in x-y plane. For the TM polarizeation the magnetic field within the cylinder has two non-zero components: H_x and H_y. To measure them both the probe loop first was aligned vertically, parallel to the y-z plane, then parallel to the x-z plane. To find the net field intensity the square of the modula of the two measured components were summed in each scanning point.

The same near-filed scanning for both polarizations was done for the empty cylinder (without the water) to measure the fields of the incident wave. To get the value of the filed enhancement the measured magnetic fields in the cylinder with the water was normalized on the measured fields of the incident wave. The obtained data are depicted in Fig. 2 and Fig. 3 of the manuscript for the TE and TM polarizations, respectively.

C. Analysis of the lineshape profile

Here we present the explicit expressions for the resonant line shape $|d_\ell(x)|^2$ and $|c_\ell(x)|^2$, following from the extension of the results of Ref. [5] to the problem in question:

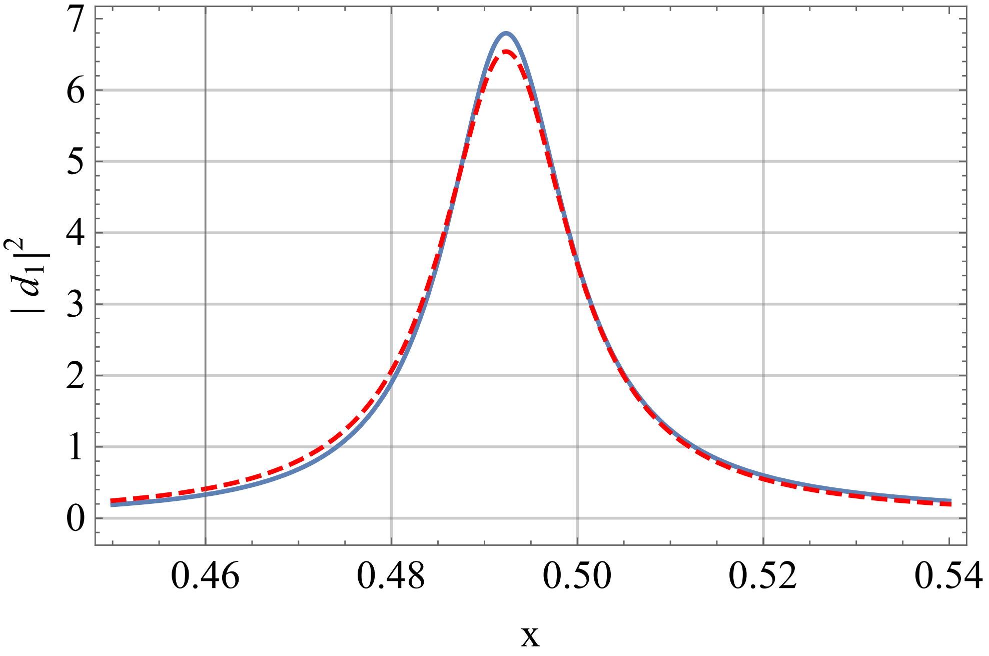

SUPPL. FIG. 3: Comparison of the exact profile $|d_1(x)|^2$, calculated according to Eq. (5) of the main text (blue solid) at $\varepsilon = 58 + 1.15i$ with the corresponding approximate Lorentzian profile, calculated according to Eq. (SI.1) — red dashed.

$$|d_\ell|^2 = \left|\frac{H_\ell^{(1)\prime}(x_{TE}^{(0)})}{J_\ell'(nx_{TE}^{(0)})}\right|^2 \frac{1}{\left(n^2\delta x - A_\ell^{(d)}(x_{TE}^{(0)})\right)^2 B_\ell^{(d)2}(x_{TE}^{(0)}) + \left(1 + \kappa B_\ell^{(d)}(x_{TE}^{(0)}) n x_{TE}^{(0)}\right)^2}, \tag{SI.1}$$

$$|c_\ell|^2 = \left|\frac{H_\ell^{(1)}(x_{TM}^{(0)})}{J_\ell(nx_{TM}^{(0)})}\right|^2 \frac{1}{\left(\frac{J_\ell''(nx_{TM}^{(0)})}{J_n(nx_{TM}^{(0)})} n^2\delta x - A_n^{(c)}(x_{TM}^{(0)})\right)^2 B_\ell^{(c)2}(x_{TM}^{(0)}) + \left(1 + \kappa \frac{J_\ell''(nx_{TM}^{(0)})}{J_n(nx_{TM}^{(0)})} B_\ell^{(c)}(x_{TM}^{(0)}) n x_{TM}^{(0)}\right)^2}, \tag{SI.2}$$

where $\delta x \equiv x - x_{TE\,(TM)}^{(0)}$, respectively;

$$A_\ell^{(d)}(x_{TE}^{(0)}) \equiv \mathrm{Re}\left[\frac{H_\ell^{(1)}(x_{TE}^{(0)})}{H_\ell^{(1)\prime}(x_{TE}^{(0)})}\right];\ B_\ell^{(d)}(x_{TE}^{(0)}) \equiv \frac{\pi x}{2}\left|H_\ell^{(1)\prime\,2}(x_{TE}^{(0)})\right|; \tag{SI.3}$$

$$A_\ell^{(c)}(x_{TM}^{(0)}) \equiv \mathrm{Re}\left[\frac{H_\ell^{(1)\prime}(x_{TM}^{(0)})}{H_\ell^{(1)}(x_{TM}^{(0)})}\right];\ B_\ell^{(c)}(x_{TM}^{(0)}) \equiv \frac{\pi x}{2}\left|H_\ell^{(1)2}(x_{TM}^{(0)})\right|; \tag{SI.4}$$

and the identities

$$\left|H_\ell^{(1)\prime\,2}(x)\,\mathrm{Im}\left[\frac{H_\ell^{(1)}(x)}{H_\ell^{(1)\prime}(x)}\right]\right| \equiv \left|H_\ell^{(1)2}(x)\,\mathrm{Im}\left[\frac{H_\ell^{(1)\prime}(x)}{H_\ell^{(1)}(x)}\right]\right| \equiv \frac{2}{\pi x} \tag{SI.5}$$

have been employed. The identities are derived exactly in the same manner as that discussed in Appendix of Ref. [5].

Eqs. (SI.1)–(SI.2) seems complicated. However, actually, they are nothing but the conventional Lorentzian profiles with cumbersome coefficients.

The high accuracy of Eqs. (SI.1)–(SI.2) should be stressed. As an example, in Suppl. Fig. 3 the exact profile $|d_1(x)|^2$, calculated according to Eq. (5) of the main text, is compared with approximate profile (SI.1) in the vicinity of the dipole resonance for the TE polarization discussed in the main text ($\varepsilon = 58 + 1.15i$) — the two profiles, practically, coincide with each other.

References

[1] W.J. Ellison, *Permittivity of pure water, at standard atmospheric pressure, over the frequency range 0 - 25 THz and the temperature range 0 - 100° C*, J. Phys. Chem. Ref. Data, **36**, 1 (2007).

[2] U. Kaatze, *Complex permittivity of water as function of frequency and temperature*, J. of Chem. and Engineering Data, **34**, 4, (1989).

[3] https://www.speag.com/products/dak/dielectric-measurements/

[4] H. Whiteside & R. King, *IEEE Trans. Antennas and Propag.*, **12**, (1964).

[5] M. I. Tribelsky & A. E. Miroshnichenko, Phys. Rev. A, **93**, 053837 (2016).
